# Supplementary material for: Pre-pandemic SARS-CoV-2 serological reactivity in rural malaria-experienced Cambodians
Source: medRxiv. 2021 Sep 28:2021.09.27.21264000. Preprint. [Version 1] doi: 10.1101/2021.09.27.21264000 (PMC8491862; doi:10.1101/2021.09.27.21264000)
Supplement: Supplement 1 [file media-1.pdf]

Appendix material

Manning et al., **Title: Pre-pandemic SARS-CoV-2 serological reactivity in rural malaria-experienced Cambodians**

Appendix Table 1. Participants' SARS-CoV-2 Seroreactivity by Site, Year, Gender and Age.

Site	Samples	Year	Males	Female	<10 years	10-17 years	>18 years
Preah Vihear	81 (15)	2011	51 (9.7)	30 (5.7)	7 (1.6)	27 (6.0)	47 (10.5)
Pursat	347 (66)	2005	261 (49.4)	86 (16.3)	20 (4.5)	71 (15.9)	256 (57.3)
		2009					
		2010					
		2011					
Ratanakiri*	100 (19)	2011	11 (2.1)	8 (1.5)	6 (1.3)	7 (1.6)	6 (1.3)
**Total**	**528**	**528**	**323 (61.2)**	**124 (23.5)**	**33 (7.4)**	**105 (23.5)**	**309 (69.1)**

All values n (%) unless otherwise noted.

*81 samples missing gender and age data

Appendix Table 2. Seropositivity status by two SARS-CoV-2 IgG assays.

Subject number	Test 1 Spike / RBD 2 S.D. cutoff	Mean RBD	Mean Spike	Spike S1-RBD commercial<br>Mean OD450 / Cutoff > 1	Mean OD450	Mean OD450 / Cutoff > 1
1	Negative	0.2157	0.2637	Negative	0.0000	0.0000
2	Negative	0.0813	0.1241	Negative	0.0005	0.0041
3	Negative	0.0847	0.3078	Negative	0.0220	0.1812
4	Negative	0.0480	0.0532	Negative	0.0077	0.0630
5	Negative	0.0854	0.5203	Negative	0.0081	0.0663
6	Negative	0.0616	0.1983	Negative	0.0221	0.1820
7	**Negative**	**0.0613**	**0.1358**	**Positive**	**0.2514**	**2.0708**
8	Negative	0.0369	0.0361	Negative	0.0215	0.1767
9	Positive	3.7444	0.7881	Positive	1.0889	8.9691
10	Negative	0.1912	0.2379	Negative	0.0112	0.0923
11	**Negative**	**3.8750**	**0.4312**	**Positive**	**0.6211**	**5.1161**
12	**Negative**	**3.8917**	**0.4280**	**Positive**	**0.6598**	**5.4349**
13	**Negative**	**3.8971**	**0.3964**	**Positive**	**0.7262**	**5.9815**
14	**Negative**	**3.0869**	**0.3910**	**Positive**	**0.4061**	**3.3451**
15	**Positive**	**3.8931**	**0.9565**	**Negative**	**0.0825**	**0.6796**
16	**Positive**	**3.8532**	**0.9306**	**Negative**	**0.0925**	**0.7615**
17	Positive	2.5646	0.7263	Positive	0.2392	1.9703
18	Positive	3.8824	1.9920	Positive	0.4771	3.9296

19	Positive	3.4743	0.9039	Positive	0.5926	4.8814
20	**Negative**	**2.2409**	**0.6454**	**Positive**	**0.2270**	**1.8694**
21	Positive	3.8911	1.9976	Positive	0.6885	5.6713
22	Negative	0.2088	0.2645	Negative	0.0517	0.4255
23	Negative	0.2221	0.1648	Negative	0.0071	0.0585
24	Negative	0.1635	0.1754	Negative	0.0089	0.0729
25	Negative	0.2032	0.2628	Negative	0.0428	0.3526
26	Positive	3.9031	1.5984	Positive	1.3099	10.7900
27	Negative	0.2109	1.0731	Negative	0.1066	0.8777
28	Negative	0.2123	0.2391	Negative	0.0813	0.6697
29	Negative	0.1325	0.1466	Negative	0.0050	0.0412
30	Positive	3.7932	0.9603	Positive	0.6937	5.7142
31	Positive	2.3932	1.5027	Positive	0.2116	1.7430
32	Negative	0.2703	0.3878	Negative	0.1121	0.9234
33	Negative	0.2216	0.3375	Negative	0.0015	0.0124
34	Negative	3.6172	0.4099	Negative	0.0972	0.8007
35	Positive	3.1024	0.7545	Positive	0.6504	5.3575
Cutoff					0.1215	
Negative control				Negative	0.0014	0.0111
Positive control				Positive	2.0219	16.6544
Positives = mean OD 450/cutoff >1						

Appendix Table 3. SARS-CoV-2 Surrogate Virus Neutralization Test (sVNT) Kit results.

Subject number	Mean OD sVNT assay	Mean / Postive CTRL	(1-Mean/Postive CTRL)	(%) Neutralizing capacity	Detectable SARS-CoV-2 neutralizing antibody	Mean RBD	Mean Spike	SPIKE / RBD seropostitive
1	2.9965	1.3873	-0.3873	-38.73	NO	0.2157	0.2637	Negative
2	2.9837	1.3814	-0.3814	-38.14	NO	0.0813	0.1241	Negative
3	3.0597	1.4166	-0.4166	-41.66	NO	0.0847	0.3078	Negative
4	3.0404	1.4076	-0.4076	-40.76	NO	0.048	0.0532	Negative
5	2.8734	1.3303	-0.3303	-33.03	NO	0.08545	0.5203	Negative
6	2.9994	1.3886	-0.3886	-38.86	NO	0.06165	0.1983	Negative
7	3.0158	1.3962	-0.3962	-39.62	NO	0.0613	0.1358	Negative
8	2.8516	1.3202	-0.3202	-32.02	NO	0.03685	0.0361	Negative
9	2.2910	1.0607	-0.0607	-6.07	NO	3.74435	0.7881	POSITIVE
10	2.8065	1.2993	-0.2993	-29.93	NO	0.1912	0.2379	Negative
11	2.2671	1.0496	-0.0496	-4.96	NO	3.87495	0.4312	Negative
12	2.3722	1.0982	-0.0982	-9.82	NO	3.8917	0.428	Negative
13	2.5024	1.1585	-0.1585	-15.85	NO	3.8971	0.3964	Negative
14	2.5129	1.1634	-0.1634	-16.34	NO	3.08685	0.391	Negative
15	2.4956	1.1554	-0.1554	-15.54	NO	3.89305	0.9565	POSITIVE
16	2.5930	1.2005	-0.2005	-20.05	NO	3.85315	0.9306	POSITIVE
17	2.2649	1.0486	-0.0486	-4.86	NO	2.56455	0.7263	POSITIVE
18	2.5655	1.1878	-0.1878	-18.78	NO	3.8824	1.992	POSITIVE
19	2.2631	1.0477	-0.0477	-4.77	NO	3.47425	0.9039	POSITIVE
20	2.4560	1.1371	-0.1371	-13.71	NO	2.2409	0.6454	Negative
21	1.8539	0.8583	0.1417	14.17	NO	3.8911	1.9976	POSITIVE
22	2.8783	1.3326	-0.3326	-33.26	NO	0.2088	0.2645	Negative
23	2.8563	1.3224	-0.3224	-32.24	NO	0.2221	0.1648	Negative
24	2.6625	1.2326	-0.2326	-23.26	NO	0.1635	0.1754	Negative
25	2.7740	1.2843	-0.2843	-28.43	NO	0.20315	0.2628	Negative
26	2.4614	1.1396	-0.1396	-13.96	NO	3.90305	1.5984	POSITIVE
27	2.8388	1.3143	-0.3143	-31.43	NO	0.21085	1.0731	Negative
28	2.7149	1.2569	-0.2569	-25.69	NO	0.2123	0.2391	Negative
29	2.6724	1.2373	-0.2373	-23.73	NO	0.13245	0.1466	Negative
30	2.2980	1.0639	-0.0639	-6.39	NO	3.79315	0.9603	POSITIVE
31	1.9825	0.9178	0.0822	8.22	NO	2.39315	1.5027	POSITIVE
32	2.1600	1.0000	0.0000	0.00	NO	0.27025	0.3878	Negative
33	2.8539	1.3213	-0.3213	-32.13	NO	0.22155	0.3375	Negative
34	2.4904	1.1530	-0.1530	-15.30	NO	3.6172	0.4099	Negative

35	2.3534	1.0895	-0.0895	-8.95	NO	3.10235	0.7545	POSITIVE
Neg. CTRL	2.5788	1.1939	-0.1939		NO			
Pos. CTRL	0.0863	0.0399	0.9601	96.01	YES			
SARS-CoV-2 Neutralizing Antibody Standard serial dilution								
156.25	1.1358	0.5258	0.4742	47.42	YES			
312.5	0.5059	0.2342	0.7658	76.58	YES			
625	0.1636	0.0757	0.9243	92.43	YES			
1250	0.0687	0.0318	0.9682	96.82	YES			
2500	0.0490	0.0227	0.9773	97.73	YES			
5000	0.0465	0.0215	0.9785	97.85	YES			
10000	0.0517	0.0239	0.9761	97.61	YES			

Appendix Figure 1. Microneutralization of SARS-CoV-2 by pre-pandemic Cambodian sera

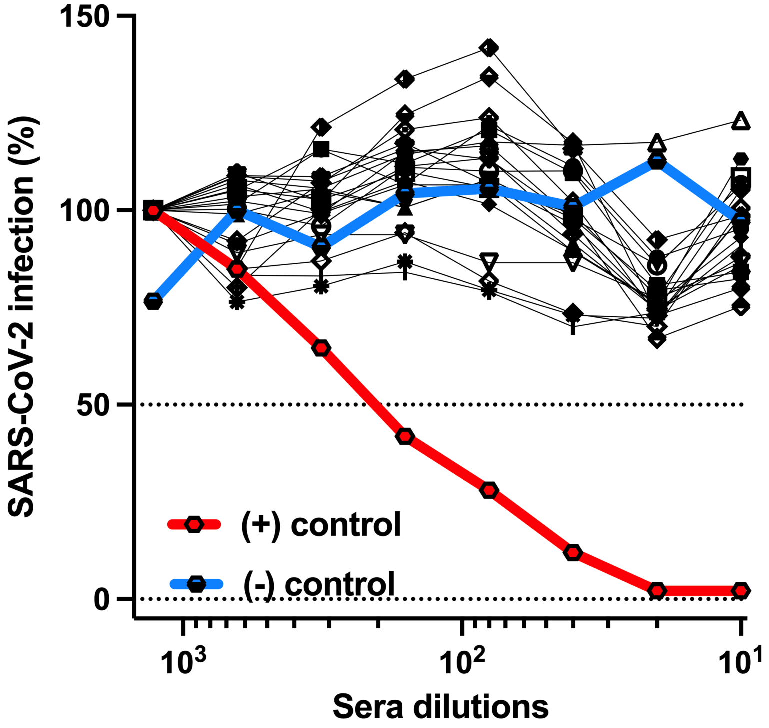

Appendix Figure 1 Legend. Twenty-one SARS-CoV-2 ELISA-positive Cambodian serum samples, negative controls (sera from US patients in 2014), and positive controls (sera from US patients who succumbed to SARS-CoV-2 infection in 2020) were heat-inactivated (56°C, 1hr), serially 2-fold diluted (1:10 to 1:1,280) in OptiPRO SFM (catalog no. 12309-019, Thermo Fisher) supplemented with 2mM L-Glutamine (catalog no. 25030-081, Thermo Fisher) and 1x Antibiotic-Antimycotic (catalog no. 15240062, Thermo Fisher). Diluted serum samples were mixed with an equal volume of SARS-CoV-2 diluted to 200 TCID50/25µl (USA-WA1/2020, catalog no. NR-52281, BEI resources) and incubated at room temperature for 1 hr. Fifty micro liter of the virus-plasma mixture was added in triplicate to Vero cells grown in a 96-well plate and incubated for 3 days at 37°C in a humidified incubator with 5% CO2. After incubation, media was removed and 200µl of 10% neutral buffered formalin (NBF) was added and incubated at room temperature for 30 min to inactivate the virus and fix the cells. After incubation, the plates were washed three times with wash buffer (0.05% Tween 20 in PBS), and 1:4,000 diluted SARS-CoV-2 nucleocapsid antibody (catalog no. 40143-R001, Sino Biological) was added to each well (50 µl per well). After incubation (room temperature, 1hr), the plates were washed 3 times, and 1:10,000 diluted horseradish peroxidase (HRP)-conjugated anti-rabbit IgG antibody (catalog no. 32460, Thermo Fisher) was added (100 µl per well), and the plates were incubated at room temperature for 1 hr. The plates were then washed 6 times followed by 30 min of room temperature incubation with HRP substrate solution (100 µl per well) prepared by adding a 10-mg o-phenylenediamine dihydrochloride tablet (catalog no. P8287, Millipore Sigma) to 20 ml of phosphate citrate buffer preparation (catalog no. P4922, Millipore Sigma). The reaction was stopped by adding 1 M sulfuric acid (100 µl per well), and the optical density was measured at 492 nm (OD492). To calculate percent infection, the optical density obtained with the lowest amount of serum (1:1,280) was used to set 100% infection for each serum sample.
